# The effect of a combined compression-tactile stimulating sock on postural stability

**DOI:** 10.3389/fspor.2024.1516182

**Published:** 2024-12-16

**Authors:** Ashleigh Marchant, Sarah B. Wallwork, Jeremy Witchalls, Nick Ball, Gordon Waddington

**Affiliations:** ^1^ Research Institute for Sport and Exercise, University of Canberra, Canberra, ACT, Australia; ^2^ IIMPACT in Health, University of South Australia, Adelaide, SA, Australia

**Keywords:** compression garment, cutaneous feedback, tactile sensation, sensory organization test, postural stability, balance

## Abstract

Previous research has demonstrated that postural stability may be improved by increasing stimulation to the somatosensory system. Wearing lower limb compression garments or textured in-soles have been found to be effective short-term methods for improving postural stability, hypothesized to be due to enhanced tactile feedback. The aim of this study was to assess whether a combined compression-tactile sock increases postural stability in healthy adults, compared to barefoot. Participants completed a sensory organization test (SOT) to assess postural stability under two conditions: (a) barefoot, and (b) wearing a compression sock with a textured inner lining (small rubber nodules on the skin side of the sole). SOT composite scores and three sensory scores - somatosensory, vestibular, visual - were assessed between the two conditions to identify whether wearing the socks was associated with enhanced postural stability. Comparisons between the two conditions were analyzed via a paired *t*-test for the (i) entire group, and an ANOVA when the group was split into (ii) “high performers” and “low performers”, according to their baseline performance on the SOT. Fifty-four participants (28 females, 26 males, mean age 40 ± 14 years) completed the study. SOT scores were not different between the compression-tactile sock and barefoot conditions when analyzed as an entire group (*p* > 0.0125), or when the group was split into performance groups (*p* > 0.0125). These findings demonstrate that, for healthy adults, mixed compression and tactile stimulation socks do not appear to be associated with improved postural stability, when measured using the sensory organization test. Although prior research indicates that wearing a compression-tactile sock improves somatosensory acuity compared to being barefoot, these benefits do not seem to carry over to postural stability. It may be that in healthy adults, the additional sensory feedback becomes redundant, or the SOT is not challenging enough for this study population.

## 1 Introduction

Balance can be described as the maintenance of postural equilibrium to prevent falling and is achieved through integration of three main afferent sensory systems: visual, vestibular, and somatosensory (1–3). These systems collaborate to form vertical alignment of body segments through continuous adjustments of muscles and joints (2, 4, 5). Postural stability is often used to describe the act of maintaining balance. Reduced postural stability and therefore poor balance can significantly impact an individuals' quality of life by increasing the risk of falls and injuries (4, 6).

Tactile feedback is one component of the somatosensory system and refers to the sensations derived from mechanoreceptors, namely myelinated Aβ fibers, within the skin which is important for balance (7, 8). Regulatory muscle movements necessary for maintaining postural stability heavily rely on tactile information received from the plantar surface of the foot (7). The plantar foot serves as the interface between the body and ground, providing information about body position (7, 8). The mechanoreceptors are sensitive to skin deformation allowing changes in pressure under the sole to provide information about foot-ground contact and whether muscular adjustments are needed to maintain posture (7, 8). These mechanoreceptors are particularly responsive to exteroceptive stimuli, such as the surface texture one is standing on, rather than internal models of equilibrium (9). Enhancing stimulation to this area increases sensory input to the central nervous system for processing and may improve balance control and postural stability (4, 7). This can be achieved through vibration and electrical stimulation to the sole of the foot, or via non-electrical stimulation such as skin indentation from a textured surface (7). Vibration applied to the sole of the foot has shown varied effects. One study found it improved balance, but only among older adults aged between 71 and 84 years compared to younger adults aged between 22 and 29 years (10), while another study reported no improvement in postural stability for both young adults (20–35 years) and older adults (70–85 years) (11). In contrast, skin indentation from a textured surface has been shown to improve postural stability among young and older adults (12). Notability (12), reported that older adults (61–80 years) increased their postural instability after the textured surface was removed, compared to the younger group (21–32 years). Using enhanced tactile stimulation to improve postural stability may be beneficial in several contexts, such as to reduce risk of falls in older adults (13, 14), for people with reduced stability due to a medical condition e.g., Parkinsons disease (15), or in situations where lower limb muscle fatigue - such as that resulting from repetitive maximal effort muscle contractions - has led to increased postural sway and reduced balance during single leg stance (16).

One simple and non-invasive way to enhance tactile feedback is through modifying footwear (4). Minimalist footwear, which promotes greater sensory contact with the ground compared to heavier shoes or boots, has been shown to improve balance performance by reducing postural sway and achieving higher balance scores compared to open type, loose shoes (4) or heavier, combat boots (17). Improved balance is often evident immediately after changing into the minimalist footwear, following a one-mile walk, and after a maximal effort load carrying task, suggesting that enhancing tactile stimulation via different footwear can be beneficial in various aspects of life. Additionally, textured in-soles have demonstrated effectiveness in reducing postural sway compared to smooth in-soles (12, 18) and enhancing dynamic balance compared to shoes with their usual manufacturer underfoot insert (19). However, in these studies it is unclear whether the minimalist footwear or texture-insoles may be more effective at improving balance compared to barefoot, as they only compare results to other types of footwear.

As an alternative to in-soles, lower limb compression garments can also improve postural stability compared to not wearing them (20). Compression garments provide increased tactile stimulation in a similar manner to in-soles; however, the stimulation of both cutaneous and joint mechanoreceptors occurs over a broader area, unlike in-soles which primarily target the sole of the foot (21, 22). Compression garments that extend from the ankle to the waist have been shown to improve postural stability and increase time in single leg balance tasks among athletes (23) and can reduce postural sway in healthy adults. Furthermore, when participants with a higher natural postural sway wear compression garment, they appear to demonstrate greater benefits in postural stability when compared to participants with a lower natural postural sway (24). In a study by Baige et al. (24) who assessed static and dynamic balance control with and without lower limb compression garments, it was found that participants with greater natural postural sway, improved their balance whilst wearing the compression garments. In contrast, participants with naturally minimal postural sway showed no improvement when wearing the compression garment. However, many studies which assess the effect of compression garments on postural stability, often exclude the foot or are in the context of sport, training, and athletic ability. Investigating the impact of a compression 'sock' on balance and stability could be valuable for developing novel interventions to support everyday tasks and mobility at home.

While there is research investigating the effects of plantar foot tactile stimulation or leg compression on postural stability, most studies only assess the impact of one modality (i.e., compression or tactile feedback). One study that did assess the effects of a textured compression knee high sock among elderly individuals found no difference in performance on a static balance task compared to both barefoot and wearing a normal sock (25). However, this study had a very small sample size (*n* = 8) and while the level of compression was reported, the details of the texture within the socks was unclear. It remains uncertain whether the integration of two or more stimulation techniques (e.g., a compression garment that also provides tactile stimulation) may improve postural stability in healthy adults.

While it is unclear whether the integration of tactile and compression garments may enhance postural stability, there is evidence to suggest that these types of garments can enhance somatosensory acuity in the ankle in healthy adults, when compared to being barefoot (26). However, this enhancement effect was only observed among individuals who had displayed below group average ankle somatosensory acuity, with no effect observed in individuals with above group average somatosensory acuity. Other studies that have investigated the use of single modalities, such as compression garments (22) or textured in-soles (27), have been found to improve ankle somatosensory acuity when compared to not wearing them. These studies also found that improvements in ankle somatosensory acuity were only observed among the lower performing individuals of the group, despite all participants being otherwise healthy. The authors suggest that tactile and compression garments may trigger cutaneous receptors and increase ankle positioning awareness in those with decreased joint awareness, while potentially overloading those with higher joint awareness (22, 26, 28). Investigating whether similar interventions translate to postural stability could offer valuable insights into how healthy adults, particularly those with below average postural stability, respond to additional and combined external stimuli.

The primary aim of this study was to investigate whether a combined compression-tactile sock (i.e., providing both compression and tactile stimulation) can enhance postural stability in healthy adults, when compared to barefoot. A secondary aim of this study was to investigate whether a combined compression-tactile sock demonstrated greater enhancements in postural stability among people with lower baseline postural stability when compared to people with higher baseline postural stability. We hypothesized individuals with below group average postural stability (low performers) would derive greater benefits in postural stability from wearing the compression-tactile sock, compared to individuals with above group average postural stability (high performers).

## 2 Materials and methods

### 2.1 Participants

Fifty-four participants were recruited for this study. This study formed a subset of a larger project registered with the Open Science Framework prior to data collection (Retrieved from osf.io/p8usy) (29). Based on the larger project, a sample size calculation was conducted using G*Power 131 (RRID:SCR_013726) which determined, we required at least 54 participants to achieve a statistical power of 0.80. Recruitment was via media advertisement (paper flyers, email, radio) and word of mouth. To be eligible to participate, participants were required to be between 18 and 65 years old, understand and speak English, and consider themselves healthy and unrestricted in movement. Exclusion criteria were any medical condition which may affect balance (e.g., vestibular conditions, diminished plantar foot sensation) and an inability to complete all tests. The study was approved by the University of Canberra Human Research Ethics Committee (reference number: 202312043).

### 2.2 Experimental protocol

Participants attended the laboratory for a single 60-minute session and provided written informed consent prior to any data collection. Demographic information was recorded on participant sex (as assigned at birth), age, height, and weight. Experimental assessment of postural stability via the SOT then commenced. All participants completed the SOT twice: once barefoot (Somatosensory Condition 1) and once wearing the compression-tactile sock (Somatosensory Condition 2). Due to a known learning effect on the SOT (30), the order of testing was pseudorandomized using an online random number generator (https://www.random.org/), to ensure that an approximately equal number of participants completed the SOT in the barefoot condition first and the compression-tactile sock condition first.

### 2.3 Somatosensory conditions

**Figure 1:**
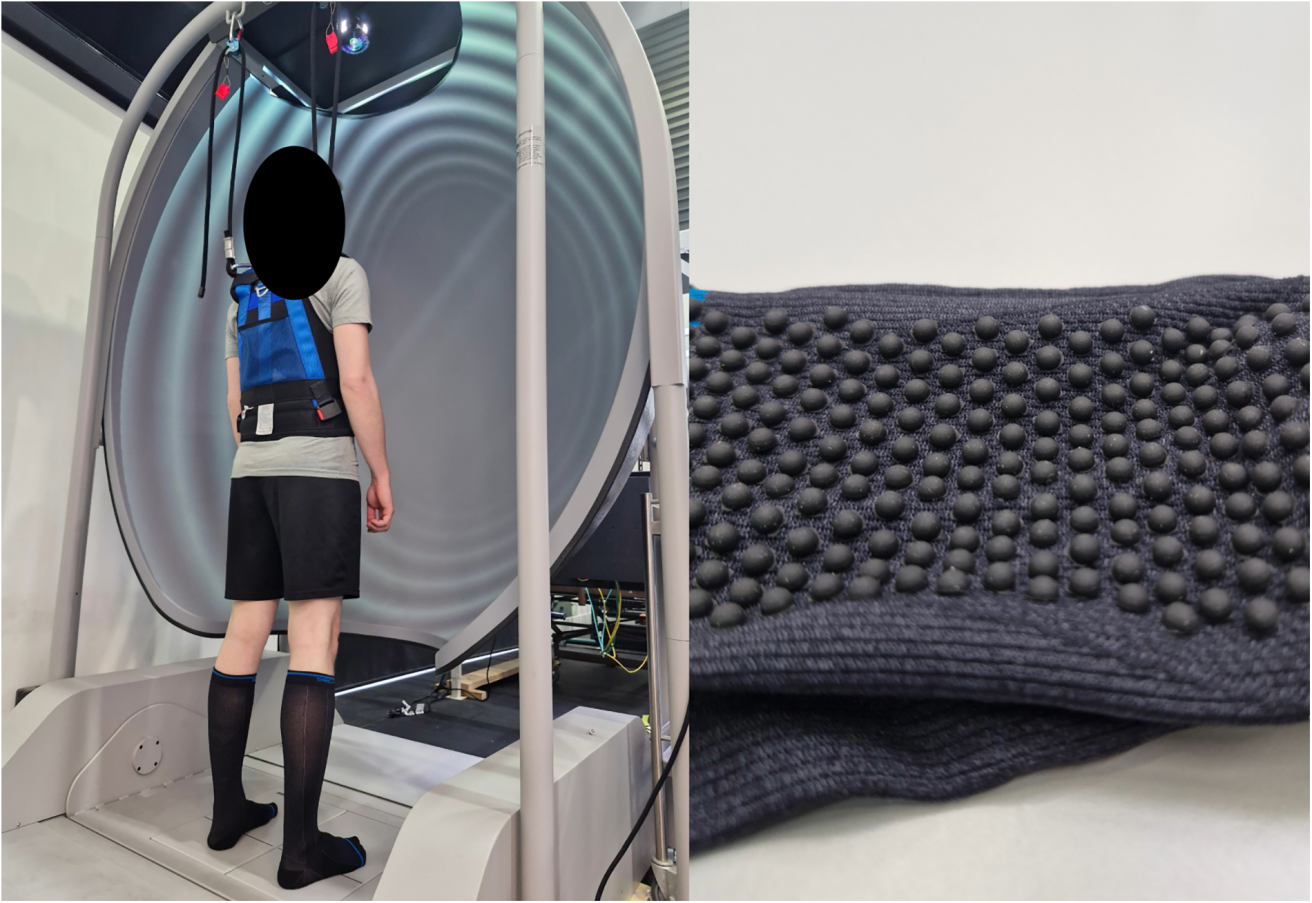
(Left) Participant completing the SOT in the compression-tactile sock condition. (Right) Close up of the compression-tactile sock. When worn, the nodules were on the inside with direct contact of the plantar foot (i.e., touching the skin on the sole of the foot).

Postural stability was measured in two conditions (1) barefoot, and (2) wearing a combined modality compression sock with a textured plantar insole (compression-tactile sock; Figure 1). The compression-tactile sock was provided by SRCHealth Pty Ltd. for the purpose of this study. The socks generated a compression rate of 20–30 mmHg and when worn, reached to approximately 50 mm below the head of fibula. Three sizes were available (small, medium, large) to ensure as much standardization as possible across participants, accommodating for individual anatomical differences. The sock had small rubber nodules attached to the inside of the sole of the sock and when the socks were worn, the rubber nodules were in direct contact with the skin of the plantar surface of the feet. Compression was applied to the foot, ankle, and distal lower leg.

### 2.4 Assessment of postural stability

Postural stability was assessed using the Sensory Organization Test (SOT) following the standardized protocol provided by the manufacturer. A Bertec® Advantage Balance System® was used to complete the SOT (Bertec Incorporated. 2014. https://www.bertec.com/. Columbus, OH, USA.). Previous research using the SOT has demonstrated poor to moderate within-session reliability, and poor between-session reliability suggesting that although the SOT is a commonly used as an assessment tool for posturography, there is a wide variance in results and learning effects occur with repeated exposure, resulting in higher scores for postural stability with repeated exposure (30). According to Wrisley et al. (31), a change of at least 8 points in the composite score is indicative of a significant intervention effect.

To complete the SOT, participants stood upon a force plate with their malleoli in line with the horizontal lines marking the rotational axis of the plate. This was the pivot point of anterior-posterior plate movement during the more challenging conditions of the test. A dome-shape screen which provided a 180-degree horizontal field of view, and a 90-degree vertical field of view was located immediately in front of the participant. A safety harness was worn throughout the assessment which only provided support should a complete loss of balance occur. Participants were asked to gaze straight ahead (in the eye-open conditions) with arms relaxed by their side.

The SOT used sway referencing capabilities via the force plates to record participants' center of pressure (COP) also known as the average area of where participants were maintaining pressure under their feet to remain in upright stance. COP is an important measure of postural stability as less sway indicates greater stability and vice versa (2). Participants undertook six test conditions which challenged their sensory systems, visual, vestibular and somatosensory, while attempting to maintain balance. Each condition was presented three times for 20 s before progressing to the next condition. In Condition 1, all sensory systems were available which acted as a marker of postural stability in quiet stance. As the test progressed, the test conditions involved minimizing input from a particular sensory system (e.g., no visual input during Conditions 1 and 5) or provided conflicting visual cues using the immersive screen (e.g., lines shifting on the screen in Condition 3) or somatosensory cues using the force plate (e.g., platform moving with shifting balance in Condition 4), or both (e.g., lines shifting on the screen and platform moving in Condition 6). See Table 1^1^ for details of the six test conditions. During the test, the force plate recorded the participants’ COP and provided a series of equilibrium scores to assess how the participant maintained postural stability throughout the tests. Equilibrium scores were calculated as a percentage that compares the participants' COP (through their feet) within the limits of postural stability (31). Thus, a score of 100% was indictive of perfect postural stability, and 0% was complete loss of balance. An equilibrium score was generated for each of the 3 tests, across the 6 conditions resulting in a total of 18 equilibrium scores.

**Table 1:** The six testing conditions of the SOT.

Condition	Vision	Surface	Visual surround	Intended sensory input
1	Eyes open	Stable	Stable	Visual, vestibular, somatosensory.
2	Eyes closed	Stable	–	Vestibular, somatosensory. Visual removed.
3	Eyes open	Stable	Sway-referenced	Vestibular, somatosensory. Visual distorted.
4	Eyes open	Sway-referenced	Stable	Visual, vestibular. Somatosensory distorted.
5	Eyes closed	Sway-referenced	–	Vestibular. Visual removed. Somatosensory distorted.
6	Eyes open	Sway-referenced	Sway-referenced	Vestibular. Visual, somatosensory distorted.

Each test was presented to participants three times for 20 s each before progressing to the next condition. Sway-reference of the platform involved a swing movement of the plate in an anterior/posterior direction, while sway-reference of the visual surround was in the form of shifting lines projected on the screen. Both were induced by the participant using feedback from the pressure under their feet. Table reprinted with permission from (31).

Scores generated by the SOT software that were used for analysis in this study included one composite score and three sensory scores. The composite score is a weighted average of the 18 equilibrium scores which accounts for the more challenging conditions by increasing the weight on test conditions 3–6 compared to test conditions 1–2 (31, 32). A high composite score (maximum of 100) indicates better postural stability compared to a low composite score (minimum of 0). The sensory scores were used to assess how much a participant relied on somatosensory, visual, or vestibular sensory feedback to maintain balance throughout the SOT tests (32). The somatosensory (SOM) score is a ratio of Condition 2 to Condition 1. The visual (VIS) score is a ratio of Condition 4 to Condition 1. The vestibular (VEST) score is a ratio of Condition 5 to Condition 1. A high sensory score is thought to indicate a greater reliance on that sensory system whereas a low sensory score is thought to indicate less reliance on that sensory system (e.g., a high SOM score closer to the maximum of 120, indicates greater use of somatosensory cues for postural stability compared to someone who scores closer to the minimum of 0).

### 2.5 Statistical analysis

SPSS statistics (IBM Corp. Released 2023. IBM SPSS Statistics for Windows, Version 29.0. Armonk, NY: IBM Corp.) was used to analyze all results. Demographic data was uploaded, and the means and standard deviations were calculated. To assess whether there was a difference between males and females in SOT composite scores, we conducted an independent samples *t*-test for barefoot and compression-tactile composite scores. To screen whether there was a learning effect between the first and second SOT assessment, regardless of somatosensory condition (barefoot vs. compression-tactile sock), we conducted a paired *t*-test between the first and second SOT test. An alpha value of 0.05 was used to determine a statistically significant result. As the order of testing was pseudorandomized, a significant result would suggest that prior exposure to the SOT had an impact to scoring instead of somatosensory condition. There were no outliers, and the data were normally distributed, as assessed by Shapiro-Wilk's test (*p* = 0.557).

To address the primary aim of the study, a paired *t*-test with a Bonferroni correction, was conducted between each SOT score (SOM, VIS, and VEST sensory score, and composite score) for the two testing conditions (barefoot vs. compression-tactile sock). Due to the multiple comparisons, an alpha value of 0.0125 was used to determine a statistically significant result. To address our secondary aim, participants were split into two postural stability groups: above group average performers (high performers) and below group average performers (low performers), based on the overall group composite data mean score. This was calculated so that individuals who had a barefoot composite score greater than the mean were considered “high performers” for this group, and individuals with a composite score below the mean were considered “low performers” for this group. This method has been used in previous ankle somatosensory acuity research and has shown differences in ankle somatosensory acuity between above and below average when wearing compression socks and compression-tactile socks (22, 26). A 2 × 2 repeated measures analysis of variance (ANOVA) with a Bonferroni correction was conducted to compare the effect of somatosensory condition (2 levels: barefoot and compression-tactile sock) for both groups (2 levels: high and low performers) on SOT performance. This was conducted for each of the SOT scores (SOM, VIS, and VEST sensory score and composite score).

## 3 Results

Fifty-four (28 female, 26 male) participants completed the study. All participants considered themselves healthy. There was no significant difference between males and females for barefoot composite scores; t(52) = −0.75, *p* = 0.456, or compression-tactile composite scores; t(52) = −1.792, *p* = 0.079. The mean and standard deviation for age, height, and weight were 40 years ± 14, 173 cm ± 9, and 76 kg ± 13 respectively. There was an overall learning effect, whereby performance improved in the second test (SOT mean composite score: 79.54) when compared to the first (first SOT mean composite score: 77.59), regardless of somatosensory condition (barefoot or compression-tactile sock); t(53) = 4.19, *p* < 0.001.

### 3.1 Effect of barefoot vs. compression-tactile socks on postural stability: entire group

There was no difference in SOT performance between barefoot and compression-tactile sock conditions for the composite score: t(53) = 0.77, *p* = 0.224; SOM sensory score: t(53) = 0.61, *p* = 0.273; VIS sensory score: t(53) = 1.22, *p* = 0.112; and VEST sensory score: t(53) = 0.31, *p* = 0.378. Table 2 presents the mean ± standard deviations of the SOT conditions.

**Table 2:** Results of the paired *t*-tests when the entire group (*n* = 54) was analyzed.

	Barefoot (M ± SD)	Compression-tactile sock (M ± SD)	*p*-value
Composite score	78.8 ± 4.4	78.4 ± 5.6	0.224
SOM sensory score	98.6 ± 2.8	98.3 ± 2.1	0.273
VIS sensory score	86.1 ± 6.4	84.8 ± 8.5	0.112
VEST sensory score	73.8 ± 9.1	73.4 ± 11.0	0.378

Test score means ± standard deviations are presented with *p* value of the comparisons. A high composite score (close to 100) indicates better postural stability while a low score (close to 0) indicates postural instability. Sensory scores (SOM, VIS, VEST) were used to assess how much participants had relied on that sensory feedback (i.e., somatosensory, visual, vestibular) to maintain balance throughout the SOT test. A higher sensory score (maximum of 120) indicates greater use of that system.

### 3.2 Comparison within high and low performing groups

**Figure 2:**
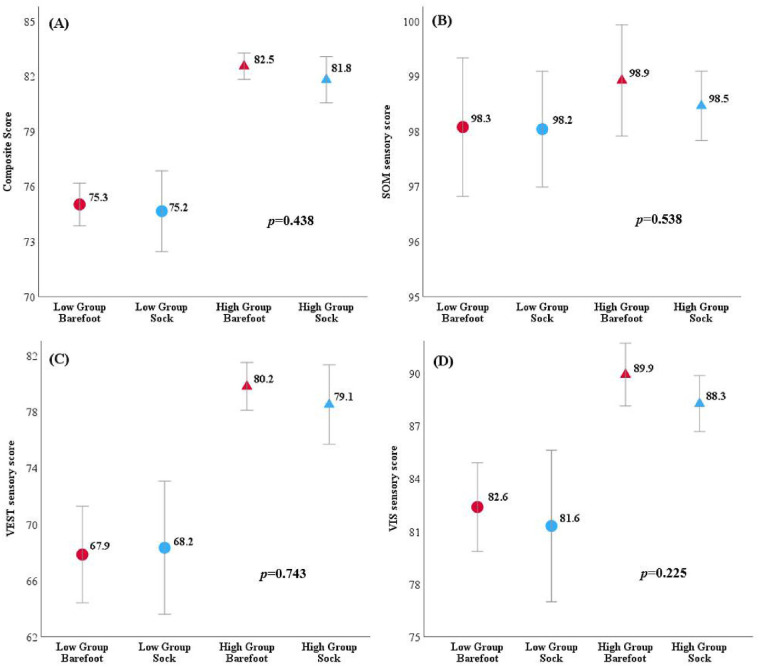
Results of the SOT scores when the data (*n* = 54) was divided into high and low performers based on the mean composite score of 78.8 for this group. There was no significant main effect (*p* value shown) between barefoot and the compression-tactile sock for both low (*n* = 28) and high (*n* = 26) performers for the **(A)** SOT composite score, **(B)** SOM sensory score, **(C)** VEST sensory score, or **(D)** VIS sensory score. The mean scores are shown where red represents the barefoot scores, and blue represents the compression-tactile sock scores. The low-performance group means are represented by circles and the high-performance group means are represented by triangles. Error bars represent the 95% confidence interval (CI).

Using the overall mean barefoot SOT composite score of 78.8, participants were grouped into low performers (barefoot score below 78.8; *n* = 28), and high performers (barefoot score of 78.8 and above, *n* = 26). Table 3 presents the demographic characteristics of the population, grouped according to performance. There was no significant main effect of wearing the compression-tactile sock compared to barefoot for both performance groups for the SOT composite scores: F(1,52) = 0.612, *p* = 0.438; SOM sensory score: F(1,52) = 0.384, *p* = 0.538; VIS sensory score: F(1,52) = 0.151, *p* = 0.225; VEST sensory score: F(1,52) = 0.108, *p* = 0.743. There was a significant main effect between the high and low performance groups for the composite score, F(1,52) = 68.459, *p* < 0.001; VIS sensory score: F(1,52) = 21.645, *p* < 0.001; VEST sensory score: F(1,52) = 4.005, *p* < 0.001 but not for the SOM sensory score: F(1,52) = 0.829, *p* = 0.367, where the high performance group had greater scores for the composite score, VIS sensory score, and VEST sensory score for both barefoot and wearing the compression-tactile sock compared to the low performing group, however were unchanged between high and low performance groups for the SOM sensory score. Figure 2 shows the means of both groups for each SOT score. There was no significant interaction between performance groups and somatosensory conditions for the composite score, F(1,52) = 0.348, *p* = 0.558; SOM sensory score, F(1,52) = 0.384, *p* = 0.652; VIS sensory score F(1,52) = 0.092, *p* = 0.225; VEST sensory score: F(1,52) = 0.298, *p* = 0.588.

**Table 3:** Performance group demographics.

Characteristic	Low performers	High performers
Sex	F 14 M 14	F 14 M 12
Age (M ± SD)	27 years ± 15	40 years ± 13
Height (M ± SD)	174 cm ± 8	171 cm ± 11
Weight (M ± SD)	75 kg ± 14	76 kg ± 12

M, mean; SD, standard deviation.

## 4 Discussion

The primary aim of this study was to investigate whether a combined compression plus tactile stimulating sock enhanced postural stability among healthy adults, when compared to barefoot. We found that compression-tactile socks did not enhance postural stability when compared to barefoot. Furthermore, the compression-tactile sock was no more beneficial for people with low baseline postural stability (low performers), when compared to people with high baseline postural stability (high performers). Our findings suggest that the combined modality (tactile and compression) sock does not appear to enhance postural stability in healthy adults when compared to barefoot.

The lack of improvement in postural stability while wearing the compression-tactile socks compared to barefoot may be because the SOT was not challenging enough for the study population of healthy adults. This theory is consistent with findings by Hatton et al. (33) who explored the effect of three types of standing surfaces on postural stability when barefoot. The authors found no difference in anterior-posterior sway, medial-lateral sway, and electromyography data of eight lower limb muscles when comparing two textured surfaces and one flat surface. Hatton et al. (33) suggest the balance test was not demanding enough for their study population (healthy adults with a mean age of 27.5 years) and the addition of the varied surfaces were meaningless. Woo et al. (20) also noted that textured materials are usually only beneficial under more challenging balance conditions such as standing with eyes closed. While the SOT does incorporate challenging conditions, such as having eyes closed (Conditions 2 and 5) or provides misleading/conflicting sensory feedback (Conditions 3–6), these conditions might not be demanding enough for healthy adults. While the protocol followed in the current study was provided by the manufacturer, if the SOT conditions had been randomized, the results might have differed. For instance, if a participant were exposed to a more challenging SOT condition first (e.g., condition 6), they may have relied more heavily on the external feedback from the sock and could have resulted in less of a learning effect. However, it is important to note that all six conditions of the SOT assess postural stability in bipedal stance, which may not provide a significant challenge to healthy adults. As a result, the compression-tactile socks might not have been able to produce a noticeable effect on postural stability in this population. Further, the current study results align with findings by Washington et al. (34) who found no improvement among healthy young adults in varying balance challenges when participants wore lower limb compression garments when compared to a sham or no garment. In the context of the SOT, footwear appears to exert the most influence on performance only after prolonged or intense physical workloads when muscle fatigue is present, with a limited effect of footwear prior to the strenuous activity (17, 35). There is mounting evidence to suggest that the sensory systems of healthy adults are already good enough to meet the demands of most tested balance tasks. This may be especially true considering the substantial sensory and tactile input received while barefoot (8), which suggests that additional sensory feedback from a garment may not further enhance postural stability.

The compression-tactile sock did not enhance postural stability in our study sample of healthy participants, perhaps because postural stability of these individuals was already high. Previous research in this field has largely been conducted on individuals with lower limb injuries, musculoskeletal conditions, or older adults. A systematic review by Woo et al. (20) highlighted that wearable garments, including compression but not textured insoles, were beneficial specifically for those with lower limb conditions such as ankle instability or knee anterior cruciate ligament reconstruction. Further, much of the research on textured materials for postural stability is primarily targeted at older adults. In both lower limb injury and older adult populations, disruption of the sensory receptors may lead to a decline in postural stability. This can potentially be mitigated by increasing stimulation to cutaneous receptors to compensate or provide more refined sensory feedback (20, 21, 36). However, in healthy individuals where sensory receptors are intact and functioning normally, additional sensory feedback may be redundant reaching a “ceiling effect” whereby there is not much room for improvement. Alternatively, the compression and textured sole components of the socks could have had conflicting effects on postural stability (i.e., the effect of one could have eliminated the effect on the other). This seems unlikely, however, because these socks have been investigated in previous work (26) demonstrating that wearing the compression-tactile socks enhanced ankle somatosensory acuity when compared to barefoot. Notably, a study by Park et al. (37) found that textured insoles did improve postural stability scores on the SOT for both healthy individuals and those with knee osteoarthritis. While their study included participants with a mean age similar to our study (52 years of age compared to our 40 years of age), Park et al. (14) specifically recruited individuals with knee osteoarthritis matched to healthy controls by age. While the minimum and maximum age range is unspecified in the study by Park et al. (37), approximately half of the participants in our study were under the age of 40 years. Globally, the prevalence of knee osteoarthritis increases above the age of 40 years and peaks at 70–79 years (38). This suggests that Park et al. (14) may have included a higher proportion of older participants compared to our study. Postural sway is shown to increase with age, with individuals aged 40–59 years showing early signs of balance deterioration compared to younger adults aged 16–39 years (39). Instead of categorizing individuals solely based on SOT performance, future studies could consider grouping participants by age to better understand age-related differences in postural stability. Alternatively, exploring the effects of compression-tactile socks in individuals with pre-existing balance-related medical or musculoskeletal conditions might provide insights into their potential benefits in clinical settings.

Our previous research has shown that wearing compression-tactile socks can enhance somatosensory acuity at the ankle (26), however the current study suggests that such improvements may not extend into enhancements in postural stability. Specifically, our earlier study found that compression-tactile socks enhanced somatosensory performance in individuals with below group average somatosensory acuity, but not in individuals with above group average somatosensory acuity. While this division of high and low performers in that study gave us further insights into who may be more likely to benefit from the compression-tactile socks, such a division of postural stability in the current study did not reveal similar group differences. It is important to note that the distinction between high and low performers in terms of postural stability in the current study may differ from that observed in our previous study, which used a division based on somatosensory acuity performance. However, it may also be that that while increasing tactile stimulation to the lower limb may benefit somatosensory acuity, it does not translate to postural stability, for which somatosensory acuity is only one contributor. Similarly, a study by Hijmans et al. (40) involving older adults found that joint position sense was enhanced with a lower limb compression bandage, compared to no bandage, but did not improve standing balance. Washington et al. (34) propose that in certain balance tasks, mechanoreceptors may not receive adequate stimulation, making any additional feedback from garments redundant. It is possible that the compression-tactile sock may improve somatosensory acuity, but such enhancements do not reach a threshold that is detectable in the SOT.

### 4.1 Limitations

This study has several limitations. First, we found a learning effect whereby participants' SOT composite scores increased during the second trial, regardless of whether they were barefoot or wore the textured-compression sock in the first task. To minimize any effects of “learning” it would have been preferable to integrate two baseline tests prior to data collection (30, 41). Secondly, the compression-tactile sock was only compared to a barefoot condition, which is a high tactile-sensory condition in and of itself. Further research investigating postural stability while wearing various tactile and compression socks might be valuable (e.g., compression-only sock, tactile-only sock, a compression-tactile sock, and a control sock.) Thirdly, the division between high and low performers on the SOT may not be a true representation of the broader population and what may be considered as high and low performance of postural stability on the SOT. However, this method did provide a strategy to further analyze how this sample responded to the additional stimuli of the compression-tactile sock.

## 5 Conclusion

We found that a mixed-modality compression-tactile sock did not improve postural stability when compared to barefoot, as measured using the Sensory Organization Test (SOT). While previous work shows that a compression-tactile sock enhances somatosensory acuity compared to barefoot, such improvements do not appear to be translated to postural stability. Future research should assess the effect of compression-tactile socks compared to barefoot or other garments, in populations in which postural stability may be reduced, such as older adults or those with musculoskeletal conditions. It is also recommended to consider completing at least two baseline SOTs to avoid potential learning effects.

## Data Availability

The raw data supporting the conclusions of this article will be made available by the authors, without undue reservation.

## Footnotes

^1^Marchant A, Witchalls J, Wallwork SB, Ball N, Waddington G. The correlation between an ankle somatosensory acuity task and sensory organisation in postural stability. (2024). [under review].
